# Electrospun Polyvinyl Alcohol Loaded with Phytotherapeutic Agents for Wound Healing Applications

**DOI:** 10.3390/nano11123336

**Published:** 2021-12-08

**Authors:** Diana Serbezeanu, Alexandra Bargan, Mihaela Homocianu, Magdalena Aflori, Cristina Mihaela Rîmbu, Alexandru Alin Enache, Tăchiță Vlad-Bubulac

**Affiliations:** 1 “Petru Poni” Institute of Macromolecular Chemistry, 41A, Grigore Ghica Voda Alley, 700487 Iasi, Romania; anistor@icmpp.ro (A.B.); michalupu@yahoo.co.uk (M.H.); maflori@icmpp.ro (M.A.); tvladb@icmpp.ro (T.V.-B.); 2 Department of Public Health, Faculty of Veterinary Medicine “Ion Ionescu de la Brad”, University of Agricultural Sciences and Veterinary Medicine, 8, Mihail Sadoveanu Alley, 707027 Iasi, Romania; crimbu@yahoo.com; 3 S.C. Apel Laser S.R.L., 25, Vanatorilor Street, Mogosoaia, 077135 Ilfov, Romania; alin.enache@apellaser.ro

**Keywords:** polyvinyl alcohol, electrospinning, *Thymus vulgaris*, *Salvia officinalis folium*, *Hyperici herba*

## Abstract

In this paper, hydroalcoholic solutions of *Thymus vulgaris*, *Salvia officinalis folium*, and *Hyperici herba* were used in combination with poly (vinyl alcohol) with the aim of developing novel poly (vinyl alcohol)-based nanofiber mats loaded with phytotherapeutic agents via the electrospinning technique. The chemical structure and morphology of the polymeric nanofibers were investigated using Fourier Transform Infrared Spectroscopy (FTIR) and Scanning Electron Microscopy (SEM). The addition of *Thymus vulgaris*, *Salvia officinalis folium*, and *Hyperici herba* extracts to the pure polyvinyl alcohol fibers led to changes in the morphology of the fibers and a reduction in the fibers’ diameter, from 0.1798 µm in the case of pure polyvinyl alcohol to 0.1672, 0.1425, and 0.1369 µm in the case of polyvinyl alcohol loaded with *Thymus vulgaris*, *Salvia officinalis folium*, and *Hyperici herba*, respectively. The adapted Folin–Ciocalteu (FC) method, which was used to determine the total phenolic contents, revealed that the samples of PVA–*Hyperici herba* and PVA–*Thymus vulgaris* had the highest phenol contents, at 13.25 μgGAE/mL and 12.66 μgGAE/mL, respectively. Dynamic water vapor measurements were used in order to investigate the moisture sorption and desorption behavior of the developed electrospun materials. The antimicrobial behavior of these products was also evaluated. Disk diffusion assay studies with *Escherichia coli*, *Staphylococcus aureus*, and Methicillin-resistant *Staphylococcus aureus* were conducted on the developed nanofibers in order to quantify their phytotherapeutic potential.

## 1. Introduction

Polymer nanofibers have enormous potential in areas such as wound healing [1,2,3], antibacterial dressings [4], biosensor devices [5,6], optoelectronics [7,8], and drug delivery systems [9,10]. Thus, nanofibers based on various polymers have attracted attention for application in the above-mentioned fields as a result of their remarkable properties, such as adjustable fiber diameter and porosity, tunable morphology, and the encapsulation capacity of the electrospun membrane. The possibility of chemical customization using various physico-chemical functionalization approaches was also taken into consideration in order to obtain improved nanomaterials. The electrospinning technique is considered to be an easy and efficient procedure to obtain nano/microfiber membranes [11]. It has been pointed out that the structural arrangement of the nanofibers obtained by the electrospinning process has been responsible for some morphological similarities that mimic the extracellular matrix (ECM) in the electrospun membranes [12]. In addition to the mentioned tunable properties, it has been established in some studies that such nanofiber membranes could be helpful for the transport of nutrients and the gas permeability of growing cells [13]. The ideal material for wound healing would help to achieve the complete healing of affected tissue and the complete restoration of its biological function, as well as preventing inflammation and microbial population [14]. In recent years, obtaining new materials and devices with applications in the fields of energy, pharmaceuticals, medicine, etc., has been investigated intensely [15,16,17,18,19]. In order to achieve this goal, studies have pursued some directions, such as identification of appropriate material, characterization of the structures obtained in terms of chemical and physical structure, investigation of morphology, cytotoxicity, water uptake/wettability, transport of water vapors through the material etc. [20,21]. The immediate dressing of a wound is considered to be the “cornerstone” in the management of its healing [22]. The essential parameters that a wound dressing material must meet are mostly related to its facile use and bio-adhesion at the injured surface, the permeability of the water vapor flux through the membrane, handy sterilization, and excellent antibacterial protection. Recently, researchers have produced several nanomaterials for rapid wound healing [23] which use herbal extracts and phytocompounds. It is well known that some plants have great potential in curing antibiotic-resistant infections [24]. It is also known that nanofiber matrices imbued with plant extracts influence wound stages such as coagulation, inflammation, wound contraction, and re-epithelialization [25]. 

Poly (vinyl alcohol) (PVA) is an extensively studied polymer that has numerous advantages—for example, enhanced hydrophilicity, non-toxicity, and biocompatibility combined with reasonable mechanical and thermal properties, gas permeability, etc. Nevertheless, for certain applications, such as hydrogel membranes for the controlled and sustained delivery of water-soluble therapeutics, it is necessary to control the balance between the hydrophilicity and hydrophobicity in this interesting polymer. While an advanced hydrophilicity of the macromolecular chain usually is responsible for a higher water solubility, which may be regarded as a cause for concern in some applications, there are many possibilities for imparting the hydrophobicity of PVA—for example, by crosslinking, copolymerization, esterification, grafting, etherification, etc. [26,27,28].

In recent years, electrospinning has been of great interest, not only because it can produce polymeric fibers with diameters between nanometers and a few micrometers using polymeric solutions or melts, but also because it has the advantage of being a simple process with which a wide range of porous structures can be produced. It is also cheap compared to conventional methods. Although electrospun PVA has been studied intensively, there are still many aspects that need to be addressed in order to further develop wound-healing materials based on PVA fibers loaded with appropriate phytotherapeutic agents.

*Thymus vulgaris* L. is an aromatic and medicinal plant that is widespread in the Mediterranean Basin. *Thymus vulgaris* L. is one of the most popular hybrid plants used worldwide and belongs to the Lamiaceae family. Since ancient times, this aromatic plant has been used for the preparation of various foods and for curing various chronic diseases. In food, *Thymus vulgaris* L. can be used fresh or dried. From a medical point of view, thyme has an antiseptic and antifungal effect. As a tea, it is useful for colds, asthma, and bronchitis as well as for treating acne. The antioxidant activity of wild thyme is mainly produced by its phenolic acids (rosmarinic acid) and flavonoids (quercitin, eriocitrin, luteolin, apigenin, and serpyllin). Carvacrol and thymol are the main phenolic components that are primarily responsible for its antioxidant activity and antimicrobial activity against a wide range of Gram-positive or Gram-negative bacteria [29]. Thymol can also be applied as an anti-inflammatory agent because it can reduce edema and diminish the affluence of leukocytes in the injured area, which is crucial for effective wound healing [30,31,32].

*Salvia officinalis folium* is widely used in popular flavors and medicines around the world. Plants of the genus Salvia, which consists of about 900 species, are generally known for their multiple pharmacological effects, such as analgesic, hepatoprotection, hypoglycemia, and anti-ischemic activities [33]. Many species of sage and their isolated constituents possess significant antioxidant activity in their enzyme-dependent and enzyme-independent systems. A number of them have shown relevant effects for the potential treatment of CNS-related disorders [33]. In addition to the antioxidant activity, many species of sage and their isolated constituents have shown anti-inflammatory, anti-nociceptive [34], and antiseptic properties [35], as well as anticancer and antimutagenic effects [33,36].

*Hyperici herba (Hypericum perforatum)* belongs to the Hypericaceae family and is recognized as one of the oldest used, most extensively investigated, and, thus, most valuable medicinal herbs in The International Pharmacopoeia [37,38]. Lately, the research interest in this herb has been renewed due to its increasing commercial value. Its well-established pharmacological properties include antiviral, neuroprotective, antifungal, anti-ischemic, wound-healing, antibacterial, antioxidant, anti-nociceptive, and antidepressant activities [39,40,41].

The main objective of the current work was the development of green polymeric nanofibrous mats based solely on PVA, without the other co-polymers or co-additives that are usually used to add value to the finite products, which are subsequently more difficult to purify and have a higher cost of production. Secondly, the PVA selected for this study had M_w_ = 30,000–80,000 Da and a degree of hydrolysis = 98%; it was chosen in order to obtain easily soluble membranes that could completely and rapidly release the phytotherapeutic agent into the environment. Hydroalcoholic solutions of *Thymus vulgaris, Salvia officinalis folium*, and *Hyperici herba* were used as phytotherapeutic agents. These were incorporated in order to endow the electrospun PVA membranes with antimicrobial activity. The PVA–phytotherapeutic fiber membranes have the advantages of rapid availability and the fact that they do not involve enormous costs. The antimicrobial and antioxidant activities of the PVA were induced by mixing the polymeric matrix with hydroalcoholic solutions of *Thymus vulgaris*, *Salvia officinalis folium*, and *Hyperici herba*.

## 2. Materials and Methods

### 2.1. Materials 

PVA powder (M_w_ = 30,000–80,000 Da, degree of hydrolysis = 98%) was bought from Sigma-Aldrich (St. Louis, MO, USA). Hydroalcoholic solutions of *Thymus vulgaris*, *Salvia officinalis folium*, and *Hyperici herba* were purchased from SC HOFIGAL EXPORT-IMPORT SA (Bucharest, Romania). The Folin–Ciocalteu reagent (Sigma-Aldrich, St. Louis, MO, USA), sodium carbonate (Sigma-Aldrich, St. Louis, MO, USA), gallic acid (Sigma-Aldrich, Darmstadt, Germany), and chromatographic grade ethanol (Merck, Darmstadt, Germany) were also purchased from commercial sources and used as received in the analyses.

### 2.2. Preparation of the Polymer Solution

The PVA solutions were prepared by dissolving a weight amount of PVA powder in distilled water at 90 °C under constant stirring for 4 h to obtain a solution with a concentration of 10 % wt./v. From the pristine solution, 4 mL was taken and 0.1 mL of hydroalcoholic solutions of *Thymus vulgaris*, *Salvia officinalis folium*, and *Hyperici herba* were added. The resulting solutions were sonicated at 50 °C for 5 min prior to being cooled down to room temperature.

### 2.3. Electrospinning Process of PVA and PVA Loaded with Phytotherapeutic Agent Solutions

The PVA and the PVA loaded with phytotherapeutic agent solutions were electrospun using a Fluidnatek^®^ LE-50 laboratory line from Bioinicia S.L. (Valencia, Spain), equipped with a variable high-voltage 0–30 kV power supply at a voltage of 20.9 kV. The distance between the tip of the needle and the collector was adjusted to 12 cm, and the flow rate of the solution was set to 10 µL/min. The electrospun fibers were collected on a foil sheet attached to a copper grid, which was used as a collector. The electrospinning process was performed for 6–8 h at room temperature and an ambient humidity of 48%.

The electrospun PVA membranes loaded with phytotherapeutic agents were sterilized under a UV lamp (254 nm) and the samples were placed in an open sterile petri dish before UV sterilization.

**Figure 1:**
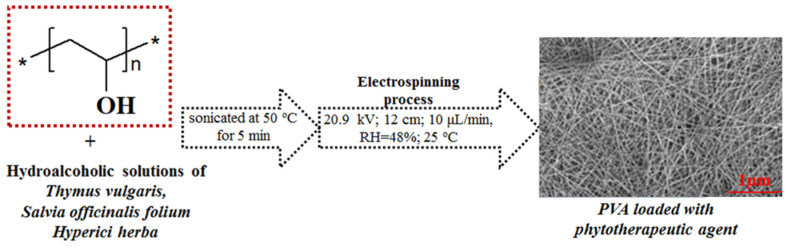
Schematic representation of the preparation of electrospun PVA membranes loaded with phytotherapeutic agents.

Figure 1 shows the schematic diagram of the preparation process of the electrospun PVA membranes loaded with phytotherapeutic agents.

### 2.4. Characterization

#### 2.4.1. FTIR Investigation

The FTIR spectra of electrospun fibers were obtained using a BioRad ‘FTS 135′ FTIR spectrometer equipped with a Specac “Golden Gate” ATR accessory. A LUMOS Microscope Fourier Transform Infrared (FTIR) spectrophotometer (Bruker Optik GmbH, Ettlingen, Germany), equipped with an attenuated total reflection (ATR) device, was used to record scans between 4000 and 500 cm^−1^ at a resolution of 4 cm^−1^.

#### 2.4.2. Membrane Morphology

The scanning electron microscopy (SEM) was performed on a TESLA BS 301 instrument at 20 kV with a magnification of 380–3600. The images were recorded on film surfaces deposed on Al supports and coated by sputtering with Au thin films using an EK 3135 EMITECH device. The diameters of the electrospun fibers were measured by means of the Image J program. At least 25 electrospun fibers from each sample were taken into consideration in order to obtain the average diameters.

#### 2.4.3. Total Phenolic Content

The total phenolic content was determined using solutions of 0.1 g nanofibers in 10 mL distilled water obtained by mixing on a magnetic stirrer at 1000 rpm and at room temperature. These solutions were used to investigate the total phenolic content using the adapted Folin–Ciocalteu (FC) method [42]. The FC reagent was diluted with ethanol/distilled water at a ratio of 1:10 (*v/v*). To prepare the sodium carbonate solution (20%), sodium carbonate (75 g/L) was dissolved in distilled water in a volumetric flask. The linear region of the gallic acid calibration curve was in the range of 6–30 µg/mL (A = −5.3342 × 10^−4^ + 0.00147X). A 2 mL sample, or standard, was mixed with 10 mL of a diluted FC reagent. After 6 min, 8 mL of 20% sodium carbonate was added and the mixture was kept in a dark place for 2 h. At the end of this period, the absorbances were recorded using an Analytik Jena 210+ spectrophotometer at a wavelength of 760 nm.

#### 2.4.4. Optical Investigation

Optical microscopy images were taken on a Zeiss Microscope Axio Imager A2M, equipped with Linkam Plate LTS420, using three different objectives.

#### 2.4.5. Dynamic Water Vapor Sorption Capacity

The dynamic water vapor sorption capacity for the samples was determined using the adapted method described previously [43].

#### 2.4.6. Profilometry

The roughness of the PVA and PVA–phytotherapeutic agent electrospun samples was investigated using a computerized high-sensitivity profilometer (Alpha–Step D 500 Stylus Profiles KLA-Tencor profile, Milpitas, CA, USA) with a vertical scanning domain of up to 1200 µm.

#### 2.4.7. Antimicrobial Activity

The antimicrobial activity testing was performed using the Kirby–Bauer disk diffusion method [44], adapted for testing PVA and PVA–phytotherapeutic agent electrospun samples. For this, the samples were cut into 5 mm disks and put into contact with three reference bacterial strains: *Staphylococcus aureus* ATCC 25923, Methicillin-resistant *Staphylococcus aureus* (MRSA) ATCC 33591 (Gram positive) and *Escherichia coli* ATCC 25922 (Gram negative). The protocol consists in the preparation of a bacterial inoculum from 24 h cultured cells with a 0.9% NaCl dilution and a turbidity of 0.5 on the McFarland scale (1.5 × 10^8^ bacterial cells/mL). The microbial culture was embedded in the culture medium Muller Hinton Agar (Oxoid), melted, and then cooled to 45 °C. After solidification, the PVA and PVA–phytotherapeutic agent electrospun disks were distributed on the surface of the culture medium with a relatively equal distance between them. The plates were then incubated under standard conditions at 37 °C for 24 h. The assessment of the antimicrobial effect was carried out qualitatively and semi-quantitatively by measuring the area of microbial inhibition created around each tested sample [44].

## 3. Results and Discussion

In this paper, we present the development of the *Thymus vulgaris–, Salvia officinalis folium–* and *Hyperici herba*–PVA electrospun scaffolds using the electrospinning method. The membrane conditions were improved by adjusting the electrospinning parameters, which included the voltage, the distance between the tip and the collector and spinner, and, of course, the pumped polymer solution. After optimization, the best conditions for voltage, tip to collector distance, and feed rate were fixed at 20.9 kV, 12 cm, and 10 μL/min, respectively. 

**Figure 2:**
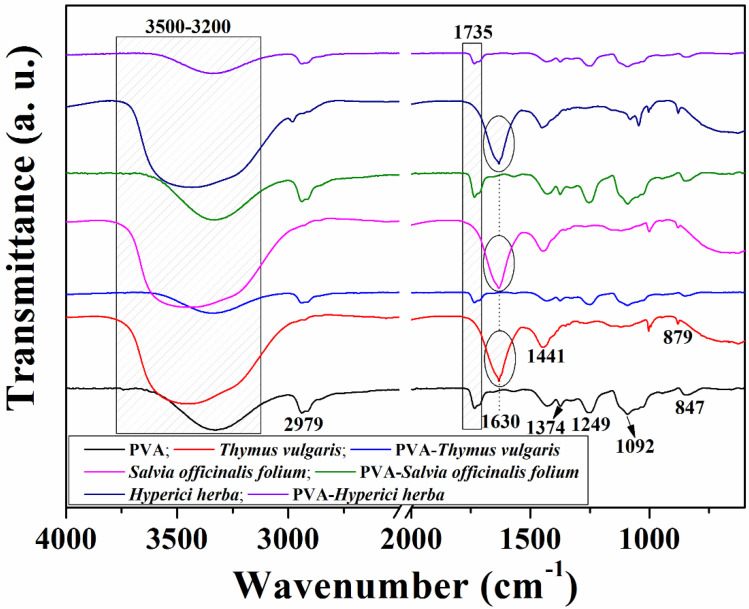
FTIR spectra of electrospun PVA fibers and PVA fibers loaded with corresponding phytotherapeutic agents in hydroalcoholic solutions.

The structure of the PVA electrospun membrane and the PVA–phytotherapeutic agents was confirmed by FTIR spectroscopy. The spectra for the PVA fibers, hydroalcoholic solutions of *Thymus vulgaris, Salvia officinalis folium* and *Hyperici herba* and the PVA fibers–phytotherapeutic agents are shown in Figure 2. The FTIR spectrum of the PVA nanofibers showed absorption bands at 1374 cm^−1^ and 847 cm^−1^ (CH, swing vibrations), 1249 and 1092 cm^−1^ (CO, tensile vibrations), and 1735 cm^−1^ (C = O, stretching vibration). In addition, there was a wide band in the range of 3200–3500 cm^−1^ with a peak at 3323 cm^−1^ and another peak at 2917 cm^−1^ due to the presence of a hydroxyl group (OH, stretching vibrations). The FTIR spectra of the hydroalcoholic solutions showed absorption bands at 3326 cm^−1^ (OH, stretching vibrations) [45] and 1441 cm^−1^ (CH, stretching vibrations, alkaloids). The absorption bands presented at 1456 cm^−1^, 1387 cm^−1^ and 1344 cm^−1^ are attributed to the phenyl ring, the plane bending vibration of OH and stretching of CO in phenol, respectively [46]. In the absorption bands at 2979 cm^−1^, 1044 cm^−1^ and 879 cm^−1^, characteristics of CH, CO and γ_C-H_ groups were observed. The components of the hydroalcoholic solutions have different groups in their structures which correspond to the absorption bands of COO, CO and phenolic groups in the region of 1750–1600 cm^−1^. The absorption band at 1456 cm^−1^, assigned to the phenyl ring in *Thymus vulgaris*/*Salvia officinalis folium*/*Hyperici herba,* was also detected in all of the PVA nanofibers loaded with phytotherapeutic agent samples, indicating the successful incorporation of phytotherapeutic agents into the PVA polymer matrix. 

**Figure 3:**
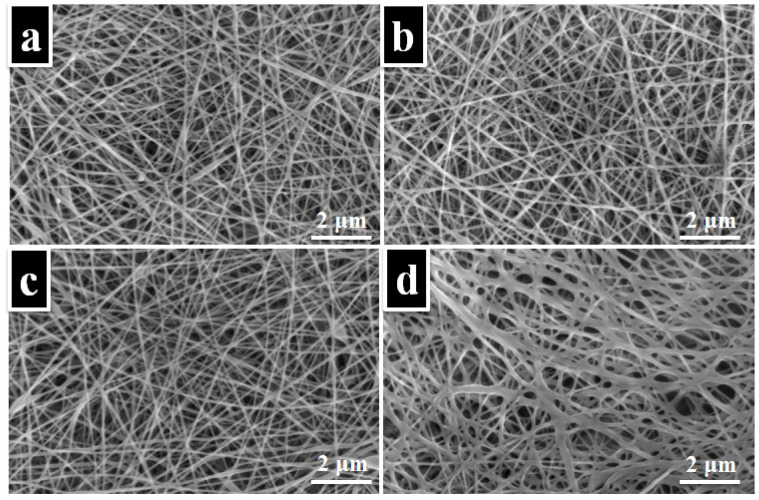
SEM images for the electrospun membranes of PVA (**a**), PVA–*Thymus vulgaris* (**b**), PVA–*Salvia officinalis folium* (**c**), and PVA–*Hyperici herba* (**d**).

Representative SEM images and the average fiber diameters for the electrospun PVA membranes are shown in Figure 3. From Figure 3, it can be observed that PVA and PVA–phytotherapeutic agents exhibited nano-scaled fibrous structures. The average fiber diameter of the PVA electrospun membrane, measured using the Image J program, was 0.1798 ± 0.05 μm. Meanwhile, the average fiber diameter distribution of PVA–*Thymus vulgari*, PVA–*Salvia officinalis folium*, and PVA–*Hyperici herba* was reduced (0.1672 ± 0.03 μm, 0.1425 ± 0.03 μm, 0.1369 ± 0.04 μm). The morphology of the PVA is quite similar to that of the PVA—phytotherapeutic agents, with no sensitive variations in the mean fiber diameter. In conclusion, we can say that the incorporation of *Thymus vulgaris* and *Salvia officinalis folium* did not affect the morphology of the electrospun membrane. When investigating the morphology of the PVA sample loaded with *Hyperici herba*, no distinct nanofibers were detected, probably due to the appearance of a certain degree of collapsing during the evaporation following the electrospinning process (Figure 3d, Table 1).

The total phenol content, expressed as the gallic acid equivalent/mL of plant extract (μg GAE/mL), of the electrospun PVA–phytotherapeutic agent fibers is shown in Table 1. All samples were analyzed in triplicate. From Table 1, it can be concluded that the sample of PVA–*Hyperici herba* had the highest phenol content (13.25 μgGAE/mL), followed by the sample of PVA–*Thymus vulgaris* (12.66 μgGAE/mL).

**Figure 4:**
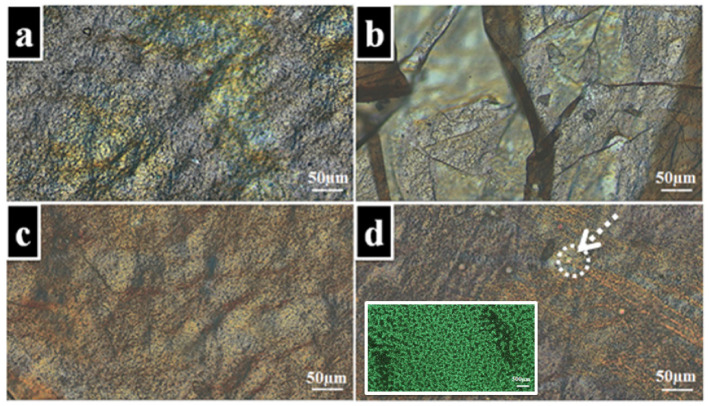
Optical microscope images of electrospun membranes PVA (**a**), PVA–*Thymus vulgaris* (**b**), PVA–*Salvia officinalis folium* (**c**), PVA–*Hyperici herba* (**d**), and *Hyperici herba* hydroalcoholic extract (d inset).

The microscopic morphology of the synthesized electrospun membranes was investigated by means of polarized optical microscopy. Microphotographs of the samples are shown in Figure 4. At an ambient temperature, all the samples featured a similar morphology in the overall microscopic view, keeping the birefringent texture of the polymer matrix (PVA) (Figure 4a) [47]. All the samples revealed a uniform dispersion of the extracts into the PVA electrospun polymer matrix, while some particularities could be observed in the morphology of the PVA–*Hyperici herba* sample as some spherical formations appeared to be relatively uniformly dispersed along the membrane under the investigation conditions. These spherical formations indicated by the arrow (Figure 4d) could be attributed to the *Hyperici herba* secretory cell [48]. The dimensions of such formations were calculated with ImageJ and found to be equal to 4.4 ± 0.56 µm. The investigation of the *Hyperici herba* hydroalcoholic extract revealed a uniform distribution along the entire microscopic view with a morphology attributed to the secretory structure of the plant, which is constituted of multiple secretory canals with dimensions of around 43.19 ± 7.49 µm (Figure 4d inset) [49]. The morphology of the PVA–*Thymus vulgaris* and PVA–*Salvia officinalis folium* samples appeared to be close to that of the neat sample without plant extracts. 

**Figure 5:**
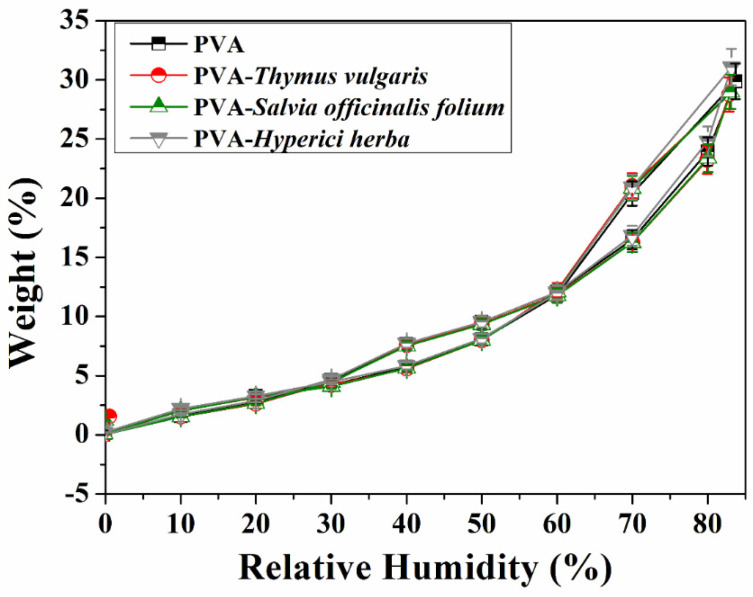
Isotherms of adsorption/dynamic desorption of water vapor of PVA fibers and PVA fibers–plant extract system.

Sorption is another interesting property, being defined as the interaction of solution (water vapor) with the adsorbent surface achieved through reversible and weak non-covalent bonds such as hydrogen bridges or van der Waals forces. The ability of the polymers to adsorb water vapor at 25 °C, in the relative humidity range 0–90%, was measured using the IGAsorp equipment. The vapor pressure was progressively increased by 10% humidity, with the vapor balance settling between 40 and 60 min. At each step, the weight accumulated at equilibrium was measured using electromagnetic compensation. The cycle was completed by progressively decreasing the vapor pressure to obtain the desorption isotherms. The samples were dried before starting the sorption experiments at 25 °C in a nitrogen stream of 250 mL/min until a constant weight of the sample was obtained at a relative humidity < 1%. The dependence between the amount of adsorbed water (wt.%) and the relative humidity for the PVA fiber sample and PVA–phytotherapeutic agents is shown in Figure 5. As can be observed from this figure, the presence of the plant extracts in the PVA fibers does not significantly influence the profile nor adsorption capacity of water vapor.

The obtained adsorption/desorption isotherms could be associated with type IV [50]; thus, the results should not be described in terms of free and bound water or hydration layers. The isotherms showed hysteresis between adsorption and desorption over two humidity ranges, 30–60% RH and 60–90% RH. In the first range, 30–60% RH, the desorption rate is higher than the absorption rate, while in the range 60–90% RH, the desorption rate is lower than the absorption rate. In a simplified manner, this can be attributed to the difference in mechanism between condensation and evaporation processes that occur on the surface of the fibers, but the network effects that may occur at the level of the fibers should not be neglected either.

In order to investigate the roughness of the square mean (R_q_) as well as the roughness of the arithmetic mean of the surface (R_a_) of the nanofibers obtained by the electrospinning process, profilometry was used. 

The R_a_ and R_q_ roughness parameters for polyvinyl alcohol (PVA) and PVA loaded with phytotherapeutic agents were in the range of 40.1–68.8 nm for R_a_ roughness (arithmetic mean roughness) and 64.2–87.7 nm for R_q_ roughness (average square roughness). Topography and morphology are essential parameters for determining the properties of the materials obtained and for the adhesion of cells to the polymeric material. It has been established that the incorporation of *Thymus vulgaris, Salvia officinalis folium* and *Hyperici herba* into the PVA nanofibers was responsible for an increase in the roughness of the PVA/extract membranes. 

**Figure 6:**
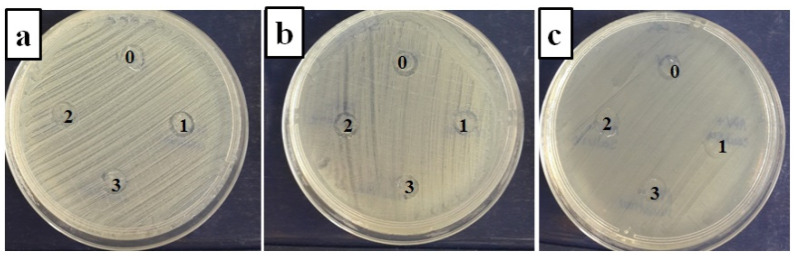
PVA electrospun membranes in contact with Staphylococcus aureus ATCC 25923 (**a**), MRSA ATCC 33591 (**b**), and *E. coli* ATCC 25922 (**c**). **0**, PVA; **1**, PVA–Thymus vulgaris; **2**, PVA–Salvia officinalis folium; **3**, PVA–Hyperici herba.

The in vitro qualitative evaluation of the microbial performance of the samples was performed by determining the average diameter of the areas of microbial inhibition which formed around the disks (5 mm) of PVA, PVA–*Thymus vulgaris*, PVA–*Salvia officinalis folium*, and PVA–*Hyperici herba* (Table 2). When introspecting the appearance of the surface of the microbial inhibition area, a central opaque area could be observed, representing the collapsed residue of the PVA–phytotherapeutic extracts, accompanied by clearer halos of inhibition formed as a result of the inhibitory action of the active principles in the biofilms. Thus, the antimicrobial effect was assessed by measuring the diameter of the created inhibition area (Figure 6).

The data obtained showed that both the neat PVA matrix and the complex samples created an inhibitory effect on all bacterial strains tested, an aspect highlighted by doubling the diameter of the inhibition zones. The antimicrobial activity results showed that plant-extract-loaded-PVA electrospun fiber mats exhibited inhibition against *Staphylococcus aureus*, methicillin-resistant *Staphylococcus aureus* (Gram positive) and *Escherichia coli* (Gram negative) bacteria. The PVA–*Salvia officinalis folium* samples had the best inhibitory activity against *Staphylococcus aureus* ATCC 25923 (11 mm) and *Escherichia coli* ATCC 25922 (11 mm); the PVA–*Hyperici herba* samples had the best inhibitory activity against *Staphylococcus aureus* ATCC 25923 (11 mm) and *Escherichia coli* ATCC 25922 (10 mm); and the PVA–*Thymus vulgaris* samples had a similar antimicrobial effect on all bacterial strains tested. Regarding the behavior of the strain *meticillin-resistant Staphylococcus aureus*, it was found that all electrospun nanofibers of PVA loaded with plant extracts inhibited bacterial cultures. This aspect is particularly important because the resistance of these strains to synthetic antimicrobials is already known [51,52]. 

These extremely important findings—namely, the capacity of these fibers to melt and to act as a whole against all tested strains—are related to the well-known properties of PVA fibers, such as biocompatibility, non-toxicity, lack of antimicrobial activity, and inhibitory effect against bacteria. Thus, although the antimicrobial effect of the matrix is strictly related to the mechanism of action of phytotherapeutic agents (polyphenols, flavonoids, etc.), no possible synergistic interrelations have been mentioned in the literature. The mechanisms of action of the plant extracts are different, acting mainly by permeabilizing the cell membranes and inhibiting the overflow pumps expressed by Gram-positive or Gram-negative bacteria [53,54], beta-lactamase inhibition [55,56], or penicillin-binding protein 2a [57].

The antimicrobial properties of *Thymus vulgaris, Salvia officinalis folium*, and *Hyperici herba* are recognized [58] and their discreet contribution in the studied samples is correlated with the quantity of hydroalcoholic solutions added to the PVA. However, the polyphenols captured in PVA fibers have damaged both gram-negative and gram-positive bacteria cells, though the wall structure of the bacterial cells of the two groups of microorganisms is different [59].

Taking into consideration the performances of the present materials and in order to initiate a path towards the commercialization and clinical implementation of such proposed biomaterials, some additional investigations need to be conducted in future studies.

## 4. Conclusions

*Thymus vulgaris, Salvia officinalis folium*, and *Hyperici herba* were successfully incorporated into PVA using the electrospinning technique. The existence of the *Thymus vulgaris, Salvia officinalis folium*, and *Hyperici herba* extracts in the PVA fibers was confirmed by FTIR. In addition, PVA–phytotherapeutic agents extract nanofibers showed potential antibacterial and antifungal capacity against *Staphylococcus aureus*, *E. coli*, and *MRSA*. The advantages of the new membrane are its simplicity, the design of a variety of sizes and shapes being entirely foldable, and its relatively low cost. The high solubility and the small average fiber diameters of the electrospun fibers make them ideal for the rapid delivery of phytotherapeutic agents, thus making possible the application of these PVA electrospun fibers in the fight against pathogenic and antibiotic-resistant microorganisms.

## Figures and Tables

**Table 1:** The values of the average fiber diameter, total phenolics, and R_a_ and R_q_ parameters of the PVA and PVA loaded with phytotherapeutic agents.

Samples	Fiber Diameter (µm)	Total Phenolics (µg GAE/mL of Extract)	Roughness Parameters	
			R_a_ (nm)	R_q_ (nm)
PVA	0.1798 ± 0.05	-	40.1	64.2
PVA–*Thymus vulgaris*	0.1672 ± 0.03	12.66 ± 0.2	50.3	73.5
PVA–*Salvia officinalis folium*	0.1425 ± 0.03	6.5111 ± 0.18	57.6	82.8
PVA–*Hyperici herba*	0.1369 ± 0.04	13.25 ± 0.26	68.8	87.7

**Table 2:** The antibacterial activity of PVA fibers and PVA fibers–plant extract system nanofibers against *S. aureus, MRSA*, and *E. coli*.

Samples		*S. aureus* ATCC 25923	*MRSA* ATCC 33591	*E. coli* ATCC 25922
Type	Ø (mm)			
		$\bar{x}$Ø (mm)	$\bar{x}$Ø (mm)	$\bar{x}$Ø (mm)
PVA	5	9	9	9
PVA–*Thymus vulgaris*	5	10	10	10
PVA-*Salvia officinalis folium*	5	11	10	11
PVA-*Hyperici herba*	5	11	10	9
